# Degree of food processing and breast cancer risk in black urban women from Soweto, South African: the South African Breast Cancer study

**DOI:** 10.1017/S0007114522000423

**Published:** 2022-12-14

**Authors:** Inarie Jacobs, Christine Taljaard-Krugell, Mariaan Wicks, Herbert Cubasch, Maureen Joffe, Ria Laubscher, Isabelle Romieu, Renata B. Levy, Fernanda Rauber, Carine Biessy, Sabina Rinaldi, Inge Huybrechts

**Affiliations:** 1 Centre of Excellence for Nutrition, North-West University, Private Bag X6001, Potchefstroom 2520, South Africa; 2 Department of Surgery, Faculty of Health Sciences, University of Witwatersrand, Private Bag X2600, Houghton, Johannesburg 2041, South Africa; 3 Non-Communicable Diseases Research Division, Wits Health Consortium (PTY) Ltd, Parktown, Johannesburg 2193, South Africa; 4 MRC Developmental Pathways to Health Research Unit, Department of Paediatrics, Faculty of Health Sciences, University of Witwatersrand, Private Bag X3, Johannesburg 2050, South Africa; 5 South African Medical Research Council, PO Box 19070, Tygerberg, Cape Town, 7505 South Africa; 6 Centro de Investigación en Salud Poblacional, Instituto Nacional de Salud Pública, CP 62100, Cuernavaca, Morelos, México; 7 Hubert Department of Global Health, Emory University, Atlanta, GA 30329, USA; 8 Department of Preventive Medicine, School of Medicine, University of São Paulo (FMUSP), São Paulo, SP 01246-903, Brazil; 9 Center for Epidemiological Studies in Health and Nutrition, University of São Paulo (Nupens/USP), São Paulo, SP 01246-904, Brazil; 10 International Agency for Research on Cancer, Nutrition and Metabolism Branch, IARC-WHO 150 cours Albert Thomas, 69372 Lyon, France

**Keywords:** NOVA, Ultra-processed, Minimally/unprocessed foods, Breast cancer risk, South Africa

## Abstract

This study aimed to investigate the association between consumption of ultra-processed foods, whole foods and breast cancer risk in black women from Soweto, South Africa. A population-based case (*n* 396)–control (*n* 396) study matched on age and residence, using data from the South African Breast Cancer study. Dietary intake was assessed using a validated quantified FFQ. Food items were categorised using the NOVA system ((1) unprocessed/minimally processed foods, (2) culinary ingredients, (3) processed foods and (4) ultra-processed foods). Conditional logistic regression models were used to estimate OR and 95 % CI of dietary contributions from each NOVA food group (as a percentage of total energy intake (EI)) and adjusting for potential confounders. Considering contributions to total EI per day, ultra-processed food consumption contributed to 44·8 % in cases and 47·9 % in controls, while unprocessed/minimally processed foods contributed to 38·8 % in cases and 35·2 % in controls. Unprocessed/minimally processed food consumption showed an inverse association with breast cancer risk overall (OR = 0·52, 95 % CI 0·35, 0·78), as well as in pre- and postmenopausal women separately (OR = 0·52, 95 % CI 0·27, 0·95 and OR = 0·55, 95 % CI 0·35, 0·89, respectively) and in women with progesterone positive breast cancer (OR = 0·23, 95 % CI 0·06, 0·86). There was no heterogeneity in association with breast cancer when analyses were stratified according to BMI. No significant associations were observed for the consumption of other NOVA food groups. Intake of unprocessed/minimally processed foods may reduce the risk of developing breast cancer in black women from Soweto, South Africa.

Breast cancer is currently the cancer with the highest incidence rate among South African women^(1,2)^. Modifiable risk factors such as an unhealthy diet, obesity and physical inactivity have already been identified and play a crucial role in primary breast cancer prevention^(3)^.

South Africa has several policies in line with the comprehensive NOURISHING (not an acronym, but a mnemonic for food policy action) policy framework of the World Cancer Research Fund and American Institute for Cancer Research, promoting healthy diets and lifestyle behaviour that may reduce the risk of developing breast cancer^(4)^. However, as a result of the nutrition transition, the South African food environment is becoming more obesogenic where whole foods are frequently being replaced by ultra-processed foods^(5,6)^. According to the NOVA food processing classification system (not an acronym but a name and hereafter referred to as the NOVA system), ultra-processed foods are defined as ‘formulations of ingredients, mostly of exclusive industry use, that result from a series of industrial processes’^(7)^. Ultra-processed foods are becoming more accessible, available and affordable in both rural and urban areas of South Africa^(8,9)^. This is worrisome since ultra-processed food consumption has been associated with an increased risk of gaining weight or becoming obese and developing non-communicable diseases, including breast cancer^(10–14)^.

Not all aspects of food processing such as grinding, milling, freezing and drying of fresh foods are nutritionally harmful and some are mainly used to manufacture various nutritious staple foods (maize meal, frozen vegetables, etc.)^(7)^. However, diets characterised by more frequent consumption of ultra-processed foods are more energy dense and contain less micro-nutrients and fibre than diets characterised with frequent consumption of unprocessed/minimally processed foods^(14–16)^.

Various classification systems have been proposed for categorising foods according to their degree of processing such as the International Food Information Council and University of North Carolina UNC systems, Foodex and LanguaL^(17–21)^. But, the NOVA system established by Monteiro and associates is used the most in research and policy development^(7,21,22)^. The NOVA system assigns foods to one of four food classification groups according to the extent and purpose of processing^(21)^. These groups include (1) unprocessed or minimally processed foods (fresh and frozen fruit and vegetables, meat, milk, etc.); (2) culinary ingredients (salt, sugar, oil and butter); (3) processed foods (smoked or cured meats, cheese, simple bread, foods preserved in salt, water or oil, etc.) and (4) ultra-processed foods (packed sweets, ready-to-eat foods, breakfast cereals, sauces, mass-produced breads, margarine, biscuits, etc.).

A previous study conducted by Jacobs and colleagues investigated the association between *a posteriori* dietary patterns and breast cancer risk in black urban South African women from Soweto. In this study, several patterns have been identified: a traditional pattern (composed of poultry, organ and offal meat, mono- and polyunsaturated fats, soup powders and vegetables), cereal-dairy breakfast-pattern (composed of milk, plain yogurt, unsweetened breakfast cereals, sorghum porridge and fruit juice, while being negatively correlated with maize meal porridge and saturated fats) and a processed food dietary pattern (composed of cheese, sweetened dairy products, candy/sugar, fast foods, alcoholic beverages, sugar-sweetened beverages, fruit spreads and crackers/potato crisps). While the traditional- and cereal-dairy breakfast dietary patterns were significantly inversely associated with breast cancer risk, no association was observed between a processed food dietary pattern and breast cancer risk^(23)^. However, this approach did not consider the degree of industrial food processing according to the NOVA system specifically. In addition, individual consumption data have never been assessed according to the degree of food processing in any South African study. Therefore, the aim of the present study was to (1) describe dietary intake of ultra-processed food according to the degree of food processing, using the NOVA system, in an urban population of Black South African women from Soweto, South Africa who participated in a population-based case–control study and (2) to investigate the association of UPF with breast cancer risk.

## Methods

### Study population

Data from the South African Breast Cancer (SABC) study, a population-based, case–control study among black urban women, were used for this study^(24–26)^. The SABC study was conducted at the Chris Hani Baragwanath Academic Hospital in Soweto from 2014 to 2017 and included a total of 399 cases and 399 matched controls. Due to missing dietary intake information, three cases and three controls were excluded. The final population of the study included 396 cases and 396 matched controls. Breast cancer cases (premenopausal *n* 133; postmenopausal *n* 248) included black urban adult women (over 18 years of age) who were newly diagnosed with breast cancer and who did not previously receive any cancer treatment. Breast cancer cases were invited to participate at referral for diagnostic biopsy, and failing that, as soon as possible after cancer diagnosis. Stage at breast cancer was clinically assessed at diagnoses and coded according to the tumour-node-metastasis classification. Breast cancer subtypes were defined using the Allred score^(24)^. Controls (premenopausal *n* 134; postmenopausal *n* 257) were black adult women from Soweto (over 18 years of age) and unrelated to breast cancer cases with no history of cancer diagnoses. Controls were recruited using a multistep sampling procedure from the areas of residency of the cases and matched by area of residence and age (±5 years)^(25,26)^. Other matching criteria of interest (e.g. reproductive factors, weight and physical activity) were captured and used in statistical models to allow us to study these criteria in relation to breast cancer risk. The sample size (396 cases and 396 matched controls) was sufficient to obtain a power > 80 % (type-II error rate *β* = 10 %) for OR_s_ ≥ 1·5 when type-I error is set to 5 %. For subcategory analysis, a size of ≥132 participants were sufficient to obtain a power > 80 % (type-II error rate *β* = 10 %) for OR_s_ ≥ 1·7 when type-I error is set to 5 %^(27)^.

### Ethical approval

The International Agency for Research on Cancer and the University of the Witwatersrand Committee for Research on Human Subjects granted ethical approval for the SABC study (M140980). The Gauteng Province Medical Advisory Committee gave permission to conduct research at the Chris Hani Baragwanath Academic Hospital. The North-West University gave ethical approval for the use of the quantified FFQ (QFFQ). All participants gave written informed consent prior to participation to the study.

### General patient information and health assessments

Trained investigators and fieldworkers conducted face-to-face interviews at inclusion in the study and for cases as soon as possible after breast cancer diagnoses. Information regarding socio-economic and demographics (income, education and other household amenities) was self-reported. Detailed information was further collected with a questionnaire regarding history of health, ethnicity, reproductive risk factors, breast health, family history of cancer, physical activity and smoking habits. Anthropometric measurements such as height, sitting height, weight and waist circumference were performed according to a standardised protocol. BMI was calculated as kg/m^2^. Questionnaires used to obtain the above-mentioned information were validated^(28–30)^.

### Dietary intake assessment

Participants were asked about their habitual dietary intake over the past month (to reduce recall bias) and dietary intake data were collected immediately after breast cancer diagnoses (at recruitment) before any cancer treatment. A validated and reproducible culture-specific QFFQ was used together with food models, food portion pictures and household utensils alongside the South African Food Composition Tables to determine habitual dietary intake^(31–33)^. The QFFQ entailed of 145 food items reported by recently published literature as regularly consumed staple foods and foods less regularly consumed. The dietary intake frequency included the amount of times foods were consumed per day/week/month or never. Life size colour photographs of thirty-seven foods (in three portion sizes) were displayed in the food portion picture booklet^(32)^. A detailed description of the method used to determine the daily intakes is described elsewhere^(34)^. The nutrient and energy intakes (EI) were calculated by multiplying the daily intake of each food item by the nutrient and energy content (per 100 g), derived from the South African Food Composition Tables, and then adding the contribution from all food items together^(35)^.

### Classifying foods based upon their level of processing

To classify foods based on the level of food processing, all foods and drinks, collected through the QFFQ, were categorised into one of the four NOVA food groups. The NOVA system was used in our study since it is the most used food classification system in research studies and will enhance comparability of our study results^(21)^. Foods were categorised into their most usual form of consumption with the most conservative classification option (less processed level) chosen in a case of doubt. Homemade dishes and culinary ingredients used in preparation methods (onions fried in oil or pumpkin candied with added sugar) were disaggregated according to the standardised recipes of the South African Food Composition Tables^(35)^. Disaggregated food items were then categorised based on the NOVA system. For instance, onions fried in oil were categorised as follows: onions as unprocessed/minimally processed food and oil as culinary ingredients.

Unprocessed or minimally processed foods are defined as foods that are naturally ready to consume or foods altered by minimal processing such as removal of inedible parts, drying, grinding, crushing, filtering, roasting, freezing, chilling, pasteurising, fermenting and boiling. These processing methods do not add any salt, sugar, oils or fats to the original products^(7)^. These foods included staple foods mostly consumed in South Africa (such as fresh fruits, 100 % fruit juices, fresh and frozen vegetable, legumes, rice, maize meal, pasta, fresh milk, milk powder, eggs, meat and fish, homemade dishes such as stews and soups).

Culinary ingredients (NOVA 2) are substances extracted from unprocessed/minimally processed foods or from nature, such as salt, sugar, fat and oil^(7)^. Industrial processes, such as pressing, refining, centrifuging, mining or extracting, are mainly used in food preparation, seasoning and cooking of unprocessed/minimally processed foods^(7)^.

Processed foods (NOVA 3) are defined as industrial products made with added salt, sugar or other culinary ingredients to unprocessed/minimally processed foods, using preservation methods. For instance, bottling or canning and using non-alcoholic fermentation in the case of cheeses and breads^(7)^. The aim of food processing in this group is to increase the durability of foods within the unprocessed/minimally processed food group and to make them more enjoyable by enhancing their sensory qualities. Foods is this category include: cheese, preserved fruits, vegetables and beans in brine, salted nuts or nut spreads, sweetened dairy products (contains only added sugar with no additives), beer, wine and non-ultra-processed bakery products.

Ultra-processed foods (NOVA 4) included carbonated drinks, industrial pre-packaged fortified* bread and buns, cookies, pastries, cakes, cake mixes, breakfast cereals, energy bars, margarines and spreads, instants sauces, soup powders, pre-prepared pies, pasta and pizza dishes, reconstituted meat products, sweet and savoury packaged snacks, ice-cream, fruit yogurts, instant desserts and noodles and distilled alcohol products such as whiskey, gin, rum and vodka. All breads were considered to be industrial pre-packed breads and were classified under ultra-processed foods since the QFFQ used to determine dietary intake did not specify between processed or ultra-processed bread. *Fortification of wheat flour, used to make bread, is compulsory in South Africa since bread is considered a staple food (fortified with vitamin A, riboflavin, niacin, pyridoxine, thiamine, folic acid, Fe and Zn).

### Statistical analysis

Descriptive analyses were presented by mean values and standard deviations (data which were normally distributed) and median; 25th−75th percentile (for data with skewed distributions). Differences between breast cancer cases and controls were assessed using paired sample *t* test (normal distributed data) and Wilcoxon signed rank test (skewed data) for continuous variables and paired *χ* ^2^ test for categorical variables (presented as percentages).

For each participant, the relative energy contribution (percentage kJ of total EI/d) of each NOVA food group in the diet was calculated and then categorised into tertiles. Conditional logistic regression was applied to compute OR and associated 95 % CI to determine the associations with each NOVA food group (dose–response analysis of tertiles) and breast cancer risk and when stratified by oestrogen positive and progesterone positive (PR+) receptors. Due to the limited number of participants in each breast cancer subcategory, which may have insufficient statistical power, we decided to explore associations by oestrogen positive and PR+ receptors only, and not in combination. Unconditional logistic regression was used to determine the association with breast cancer risk when analysis was stratified by menopausal status (pre *v*. post) and BMI status (BMI < 30 kg/m^2^ *v*. BMI ≥ 30 kg/m^2^).

A two-stage model was used to obtain OR and the associated 95 % CI. Confounding factors were considered as factors influencing the crude OR output by more than 10 %. The crude output was reported in model 1, taken into account the matched factors of breast cancer cases and controls (age, demographic factors). The following confounders were examined in the analysis before insertion in model 2: age (continuous), ethnicity (Zulu/Pedi/Swazi, Xhosa, Sotho, Tshwane, Venda, Tsonga and Ndebele), ‘individual income/month in South African Rand (zero income, income between R1-R3000 (± $0·1–$195) and income ≥ R3001+ (> ± $ 195), categorised based on data from this study)’, level of education (none/primary school, high school and college/postgraduate/diploma), smoking (smokers and non-smokers), height (continuous), waist circumference (continuous), total physical activity/d (≤ 16 min/d *v*. > 16 min/d, categorised based on median of current population), age at menarche (continuous), full-term pregnancy (yes/no), age at first pregnancy (≤ 24 *v*. > 24 years of age, categorised based on median age of the current population), age of menopause (≤ 48 *v*. > 48 years of age, categorised based on median age of the current population), parity (≤ 3 children *v*. > 3 children, categorised based on median number of children of the current population), ever breast-feeding (yes/no), duration of exclusive breast-feeding (months), use of exogenous hormones (hormonal birth control to avoid pregnancy such as oral contraceptives and injections or hormone replacement therapy/combined hormone replacement therapy after menopause), family history of breast cancer (yes/no), alcohol consumption, HIV positivity (yes/no) and reporting of energy (under-reporting *v*. over-reporting).

Only ethnicity, individual income per month, waist circumference (not adjusted for waist circumference when analysis was stratified by obesity status), physical activity and menopausal status (not adjusted for menopausal status when analysis was stratified by menopausal status) influenced the crude output by more than 10 % and were therefore included in model 2.

A generalised linear model was used to estimate the differences in least square means (measured in kJ per day) of (1) unprocessed/minimally processed foods, (2) processed foods and (3) ultra-processed foods between breast cancer cases and control participants. The effect of potential confounders (the same potential confounders used in the regression model) was tested for by including additional variables into the generalised linear regression model.

## Results

Table 1 presents the distribution of selected characteristics between breast cancer cases and control participants. Ethnicity differed significantly among cases and control participants with cases having more Ndebele-speaking people and controls having more Sotho-speaking people. Breast cancer cases had a significant lower waist circumference (93·3 cm (sd 13·8) cm) compared with controls (95·8 cm (sd 13·7) cm) and had a lower percentage of HIV-positive women (16·5 % *v*. 22·6 %). Considering dietary factors, the percentage of non-alcohol consumers was higher in cases (80·8 %) than in control participants (69·4 %). Additionally, in breast cancer case participants, oestrogen positive (75·2 %) and PR+ (75·2 %) were the dominant hormonal breast cancer tumour receptors, while triple negative breast cancer receptors accounted for 16·1 % of all tumour types.

**Table 1.** Distribution of selected characteristics between cases and control participants (Mean values and standard deviations, percentiles based on distribution of variables and percentages)

	Controls (*n* 396)		Cases (*n* 396)		
Characteristics	*n*	%	*n*	%	*P*
Socio-demographic factors					
Age (years)					
Mean	54·6		54·7		0·980
sd	12·9		12·9		
Ethnicity					0·041
Zulu/Pedi/Xhosa/Tswana/Swazi	66	16·6	67	16·9	
Sotho	144	36·4	108	27·3	
Venda/Tsonga	91	23·0	105	26·5	
Ndebele	95	24·0	116	29·3	
Level of education					0·078
None/primary	71	17·9	97	24·5	
High school	279	70·5	257	64·9	
College/University/postgraduate	46	11·6	42	10·6	
Individual income/month					0·350
R0	108	27·3	125	31·6	
R1–R3000 (*n*/%)	227	64·2	219	59·8	
R3001^+^ (*n*/%)	61	8·5	52	8·6	
Anthropometry					
BMI					0·790
Underweight < 18·5 kg/m^2^	5	1·3	7	1·8	
Normal weight ≥ 18·5 and ≤ 24·9 kg/m^2^	63	15·9	71	17·9	
Overweight ≥ 25·0 and ≤ 29·9 kg/m^2^	93	23·5	87	21·9	
Obese ≥ 30·0 kg/m^2^	235	59·3	231	58·4	
WC (cm)					0·011
Mean	95·8		93·3		
sd	13·7		13·8		
Lifestyle factors					
Total vigorous and moderate PA min/week					
Median	32·1		39·4		0·303
25th percentile, 75th percentile	9·1, 70·8		7·9, 85·8		
Current smokers	44	11·1	35	8·8	0·286
HIV positivity	90	22·6	65	16·5	0·025
Dietary factors					
TE (kJ/d)					
Median	8990		9146		0·239
25th percentile, 75th percentile	7184, 10 284		6812, 9759		
Protein (g/d)					0·073
Median	63·5		63·8		
25th percentile, 75th percentile	49·2, 93·1		47·4, 82·7		
% of TE	12·0		11·8		
Fat (g/d)					0·125
Median	64·4		64·8		
25th percentile, 75th percentile	47·2, 95·7		42·4, 91·9		
% of TE	27·2		26·9		
CHO (g/d)					0·445
Mean	338·7		330·8		
sd	147·3		143·5		
% of TE	64·0		61·4		
Dietary Fibre (g/d)					0·616
Mean	25·4		24·9		
sd	11·5		11·0		
Added sugar (g/d)					0·313
Median	67·9		65·3		
25th percentile, 75th percentile	39·9, 109·7		38·4, 105·5		
% of TE	12·0		12·1		
Non-alcohol consumers	275	69·4	320	80·8	<0·001
Ethanol intake (g/d)					0·005
Median	4·6		5·4		
25th percentile, 75th percentile	2·5; 14·7		2·8; 13·8		
NOVA food groups					
Unprocessed/minimally processed foods (g/d)					
Median	1619·4		1480·5		0·029
25th percentile, 75th percentile	1115·2, 2568·0		1060·8, 2032·8		
Culinary ingredients (g/d)	33·0		26·5		0·081
Median	16·7, 115·0		14·1, 77·5		
25th percentile, 75th percentile					
Processed foods (g/d)					<0·001
Median	87·1		63·9		
25th percentile, 75th percentile	28·3, 303·0		18·2, 172·9		
Ultra-processed foods (g/d)					
Median	753·5		603·9		<0·001
25th percentile, 75th percentile	340·2, 1936·4		300·1, 1155·9		
Breast cancer risk factors					
Full term pregnancy in parous women	382	100	377	100	0·374
Ever breastfed in parous women	349	91·3	339	89·9	0·293
Duration of breast-feeding (1) (months)	32	12, 60	30	8, 58	0·187
Use of birth control (contraceptives)	215	54,3	229	57·8	0·316
Postmenopausal	257	64·8	248	62·6	0·852
Age at menopause (2) (years)	48	44, 50	47	42, 50	0·331
Family history of BC	17	4·3	25	6·3	0·205
Age at menarche					
Median	15		15		0·537
25th percentile, 75th percentile	13, 16		13, 16		
Breast cancer case characteristics					
Stage at BC diagnoses					
I	–		24	6·5	
II	–		175	44·8	
III	–		161	40·8	
IV	–		31	7·9	
Receptor status					
ER+	–		298	75·2	–
PR+	–		263	66·4	–
HER2	–		114	28·8	–
Breast cancer subtype†					
HER2 enriched	–		21	5·3	–
Luminal A	–		40	10·1	–
Luminal B	–		269	67·9	–
TNBC	–		64	16·2	–

WC, waist circumference; TE, total energy; CHO, carbohydrates; PA, physical activity; ER+, oestrogen receptor positive; PR+ progesterone receptor positive; HER2, human-epidermal growth factor-2; TNBC, triple negative breast cancer; HRT, hormone replacement therapy.

Continuous variables are presented as means and standard deviations if normally distributed and median (25th percentile, 75th percentile) if not, categorical variables are presented as percentages.

* 16 Missing values for menopausal status (15 cases and 1 control). Missing values were excluded from percentage calculations

† Defined using Allred score.

(1) In breast-feeding women only.

(2) Among postmenopausal women only.

**Fig. 1.**
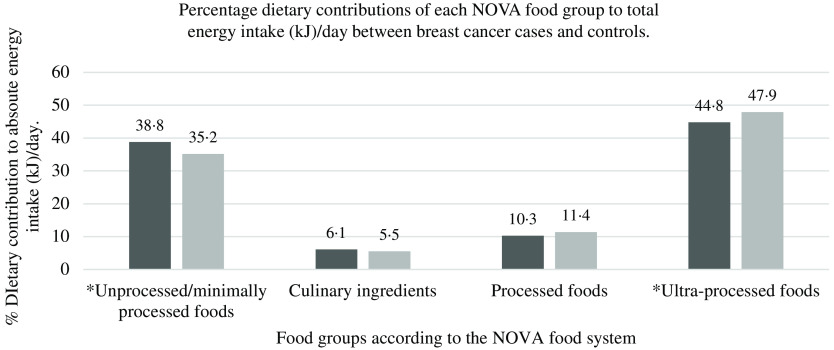
Percentage dietary contributions of each NOVA food group to total energy intake (kJ) (per day) between (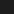) breast cancer cases and (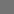) controls.

With regard to total EI per day (of habitual dietary intake), ultra-processed food consumption contributed to 44·8 % in breast cancer cases and 47·9 % in control participants (*P* < 0·05) (see Fig. 1). Unprocessed/minimally processed foods contributed to 38·8 % in breast cancer cases and 35·2 % in control participants, while processed food consumption contributed to 10·3 % in breast cancer cases and 11·4 % in control participants’ total EI per day. Culinary ingredients contributed the least to total EI/d in both breast cancer cases (6·1 %) and controls (5·5 %).

Table 2 provides the OR and 95 % CI of unprocessed/minimally processed and ultra-processed foods in association with breast cancer risk. Trend analysis of the crude model showed that higher consumption of unprocessed/minimally processed foods was inversely associated with breast cancer risk overall (OR = 0·50, 95 % CI 0·34, 0·73, *P*-trend < 0·001), for postmenopausal women (OR = 0·50, 95 % CI 0·32, 0·78, *P* = 0·003), for PR+ breast cancers (OR = 0·30, 95 % CI 0·09, 0·93, *P*-trend = 0·037) and for obese women (OR = 0·55, 95 % CI 0·35, 0·89, *P*-trend = 0·014). Trend analysis further showed that higher consumption of culinary ingredients, processed foods and ultra-processed foods was inversely associated with breast cancer risk overall (OR = 0·53, 95 % CI 0·36, 0·75, *P*-trend < 0·001, OR = 0·53, 95 % CI 0·37, 0·77, *P*-trend = 0·001 and OR = 0·47, 95 % CI 0·31, 0·72, *P*-trend < 0·001, respectively).

**Table 2.** Relative intakes (percentage of total energy intake) for each NOVA food group (dose–response analysis of tertiles) in association with breast cancer risk (Odds ratios and 95 % confidence intervals)

	Unprocessed/minimally processed foods				Culinary ingredients				Processed foods				Ultra-processed foods			
	Model 1		Model 2		Model 1		Model 2		Model 1		Model 2		Model 1		Model 2	
	OR	95 % CI	OR	95 % CI	OR	95 % CI	OR	95 % CI	OR	95 % CI	OR	95 % CI	OR	95 % CI	OR	95 % CI
Overall (cases *n* 396; controls (*n* 396)																
Lowest tertile	1		1		1		1		1		1		1		1	
Middle tertile	1·02	0·73, 1·42	1·01	0·72, 1·43	0·90	0·65, 1·26	0·84	0·59, 1·19	0·83	0·59, 1·17	0·79	0·54, 1·14	1·28	0·91, 1·83	1·36	0·89, 2·10
Highest tertile	0·50	0·34, 0·73	0·52	0·35, 0·78	0·53	0·36, 0·75	0·55	0·38, 1·32	0·53	0·37, 0·77	0·86	0·63, 1·15	0·47	0·31, 0·72	1·03	0·72, 1·45
*P*-trend	<0·001		0·001		<0·001		0·336		0·001		0·210		<0·001		0·149	
Premenopausal (cases *n* 148; controls *n* 139)*																
Lowest tertile	1		1		1		1		1		1		1		1	
Middle tertile	0·72	0·42, 1·26	0·66	0·37, 1·18	1·00	0·58, 1·72	0·94	0·54, 1·65	0·68	0·39, 1·21	0·74	0·41, 1·32	1·14	0·64, 2·05	1·25	0·68, 2·25
Highest tertile	0·56	0·31, 1·03	0·51	0·27, 0·95	0·74	0·41, 1·33	0·67	0·37, 1·24	0·43	0·23, 0·78	0·45	0·24, 1·56	0·63	0·32, 1·22	0·62	0·32, 1·22
* P*-trend	0·062		0·035		0·311		0·212		0·192		0·445		0·171		0·168	
Postmenopausal (cases *n* 248; controls *n* 257)*																
Lowest tertile	1		1		1		1		1		1		1		1	
Middle tertile	1·23	0·81, 1·86	1·26	0·83, 1·92	0·77	0·51, 1·18	0·72	0·46, 1·10	0·91	90·59, 1·38	0·90	0·59, 1·38	1·41	0·93, 2·12	1·44	0·95, 2·12
Highest tertile	0·50	0·32, 0·78	0·55	0·35, 0·89	0·45	0·29, 1·10	0·46	0·29, 1·33	0·60	0·39, 1·23	0·65	0·42, 1·03	0·48	0·30, 0·75	0·89	0·62, 1·29
*P*-trend	0·003		0·012		0·201		0·112		0·122		0·070		0·104		0·130	
ER+ (cases *n* 298)																
Lowest tertile	1		1		1		1		1		1		1		1	
Middle tertile	0·86	0·32, 2·28	0·91	0·31, 2·71	0·78	0·29, 2·09	1·03	0·34, 3·13	2·13	0·78, 5·83	2·33	0·75, 7·26	3·69	1·31, 10·41	5·41	1·66, 7·91
Highest tertile	0·52	0·21, 1·30	0·53	0·19, 1·48	0·64	0·30, 1·38	0·75	0·32, 1·73	0·71	0·32, 1·51	0·79	0·34, 1·82	1·01	0·42, 2·45	1·31	0·48, 3·57
*P*-trend	0·165		0·226		0·260		0·505		0·369		0·581		0·974		0·586	
PR+ (cases *n* 263)																
Lowest tertile	1		1		1		1		1		1		1		1	
Middle tertile	0·53	0·16, 1·71	0·52	0·14, 1·97	1·38	0·45, 4·21	1·61	0·42, 0·62	1·27	0·42, 3·81	2·29	0·56, 0·93	4·43	1·29, 6·39	8·95	1·93, 41·45
Highest tertile	0·30	0·90, 0·93	0·23	0·06, 0·86	0·71	0·31, 1·65	0·74	0·28, 1·94	0·48	0·19, 1·19	0·51	0·17, 1·55	0·89	0·33, 2·44	1·11	0·34, 3·58
*P*-trend	0·037		0·029		0·433		0·551		0·114		0·237		0·823		0·854	
BMI < 30 kg/m^2^ (cases = 165; controls *n* 161)																
Lowest tertile	1		1		1		1		1		1		1		1	
Middle tertile	1·15	0·68, 1·93	1·07	0·61, 1·87	1·31	0·77, 2·23	1·32	0·77, 2·27	1·08	0·64, 1·82	1·04	0·60, 1·81	1·25	0·75, 2·01	1·22	0·67, 2·25
Highest tertile	0·50	0·28, 0·88	0·47	0·26, 0·85	0·61	0·35, 1·05	0·58	0·33, 1·01	1·21	0·84, 1·73	0·50	0·29, 1·23	1·18	0·82, 1·71	1·01	0·59, 1·52
*P*-trend	0·117		0·014		0·074		0·056		0·310		0·872		0·354		0·516	
Obesity (cases *n* 231; controls *n* 235)*,†																
Lowest tertile	1		1		1		1		1		1		1		1	
Middle tertile	0·93	0·61, 1·43	0·89	0·57, 1·37	0·64	0·42, 0·98	0·61	0·39, 0·94	0·70	0·45, 1·08	0·70	0·45, 1·09	1·35	0·88, 2·08	1·3	0·84, 2·02
Highest tertile	0·55	0·35, 0·89	0·57	0·35, 0·92	0·48	0·31, 1·04	0·51	0·32, 1·21	0·56	0·36, 1·09	0·59	0·37, 1·03	0·64	0·40, 1·05	0·67	0·41, 1·08
*P*-trend	0·014		0·021		0·071		0·203		0·116		0·115		0·077		0·103	

ER+, oestrogen receptor positive; PR+, progesterone receptor positive.

Model 1: crude output.

Model 2: adjusted for individual income per month, ethnicity, physical activity, waist circumference (not adjusted for waist circumference when stratified by obesity status) and menopausal status (not adjusted for menopause when stratified by menopausal status).

* Unconditional logistic regression.

† Obesity defined as BMI ≥ 30 kg/m^2^.

After adjusting for possible confounders, the associations between breast cancer risk and higher consumption of unprocessed/minimally processed foods remained significant for breast cancer risk overall (OR = 0·52, 95 % CI 0·35, 0·78, *P*-trend = 0·001), for postmenopausal women (OR = 0·55, 95 % CI 0·35, 0·89, *P*-trend = 0·012) for PR+ breast cancers (OR = 0·23, 95 % CI 0·06, 0·86, *P*-trend = 0·029), for women with a BMI < 30 kg/m^2^ (OR = 0·47, 95 % CI 0·26, 0·85, *P*-trend = 0·014) and for women with a BMI ≥ 30 kg/m^2^ (OR = 0·57, 95 % CI 0·35, 0·92, *P*-trend = 0·021). An inverse association with breast cancer risk and higher consumption of unprocessed/minimally processed foods was additionally noted for premenopausal women (OR = 0·51, 95 % CI 0·27, 0·95, *P*-trend = 0·035).

No significant associations were observed between higher consumption of culinary ingredients, processed- or ultra-processed foods and breast cancer risk when adjusted for possible confounders. Sensitivity analysis was done by classifying industrial pre-packed bread under the processed food group (NOVA 3) (instead of under ultra-processed foods) in the analysis, since the QFFQ used to determine dietary intake did not specify whether breads consumed were processed or ultra-processed. However, sensitivity analysis did not significantly change the results. Sensitivity analysis was conducted, using the proportion of each NOVA food group as a weighted ratio (percentage g/d) of the total food intakes to take into account non-nutritional factors that do not provide any energy (artificial sweeteners and additives) in ultra-processed foods. Results did not differ from the analysis with food groups measured as percentage kJ/d.

Table 3 presents the nutrient profile of each NOVA food group (comparing the highest tertile of each NOVA food group). The unprocessed/minimally processed food group had the lowest total energy content (median = 7344 kJ, 5764–9172 kJ) followed by culinary ingredients (median = 9619 kJ, 7484–12 338 kJ), processed foods (median = 11 314 kJ, 8334–14 862 kJ) and the ultra-processed food group showing the highest total energy content (median = 10 982 kJ, 7561–14 958 kJ). Comparing all the NOVA food groups, the ultra-processed food group had the highest content of saturated fat (median = 22·9 g, 14·9–32·5 g) and added sugar (median = 82·3 g, 41·8–141·9 g) while having the lowest content of dietary fibre (mean = 19·7 g (sd 13·1) g). The unprocessed/minimally processed food group had the highest content of dietary fibre (mean = 28·1 g (sd 14·9) g) while having the lowest content of saturated fats (median = 14·4 g, 10·1–21·5 g) and added sugar (median = 60·8 g, 34·9–95·6 g). Both the unprocessed/minimally processed and ultra-processed food groups had similar high contents of micronutrients.

**Table 3.** Nutrient profile of each NOVA food group overall (comparing the highest tertile of each food group) (Median values and percentiles; mean values and standard deviations)

	Unprocessed/minimally processed foods		Culinary ingredients		Processed foods		Ultra-processed foods	
	Median	25th–75th percentiles	Median	25th–75th percentiles	Median	25th–75th percentiles	Median	25th–75th percentiles
Energy (kJ)*	7344	5764–9172	9619	7484–12 338	11 314	8334–14 862	10 982	7561–14 958
Total protein (g)*	74·2	56·7–95·7	62·6	47·5–82·4	79·4	55·4–105·7	72·5	52·8–107·2
Plant protein (g)*	34·8	24·9–48·5	28·8	21·8–37·9	25·1	19·7–31·7	31·8	23·8–45·3
Animal protein (g)*	37·4	25·2–52·6	31·5	21·1–45·6	40·1	26·4–56·3	38·0	23·1–57·3
Total fat (g)*	76·8	49·9–103·5	71·4	49·3–100·9	83·7	56·5–110·7	77·9	50·2–109·4
Saturated fat (g)*	14·4	10·1–21·5	17·1	11·3–25·2	22·8	16·1–31·5	21·9	14·9–32·5
Monounsaturated fat (g)*	24·2	15·9–32·7	22·3	14·6–31·7	26·5	17·9–35·1	24·4	15·9–34·4
Polyunsaturated fat (g)*	21·1	13·5–29·8	19·8	12·8–29·2	23·7	15·2–32·6	21·3	13·7–30·8
Cholesterol (g)*	198·0	125·7–282·1	252·3	151·0–399·1	330·4	192·9–490·2	286·8	166·5–432·3
Total CHO (g)†								
Mean	339·4		328·6		354·7		346·1	
sd	185·2		177·4		173·6		179·9	
Added sugar (g)*	60·8	34·9–95·6	84·1	43·4–154·4	79·2	41·9–121·1	82·3	41·8–141·9
Dietary fibre (g)†								
Mean	28·1		21·4		19·8		19·7	
sd	14·9		10·9		9·7		13·1	
Protein: CHO: Fat ratio‡	1:4·6:2·3		1:5·2:2·5		1:4·5:2·4		1:4·8:2·4	
Ca (mg)*	552·1	367·0–815·6	404·3	244·7–621·3	363·9	288·1–543·4	520·8	365·1–717·2
Fe (mg)†								
Mean	17·2		15·1		17·2		17·1	
sd	9·4		6·7		5·9		8·9	
Mg (µg)†								
Mean	352·1		309·9		319·7		344·9	
sd	195·4		143·7		119·1		171·4	
P (mg)*	1139·2	816·7–1679·8	946·7	704·7–1227·5	1068·8	815·7–1331·1	1121·8	899·9–1437·5
K (mg)*	2770·5	1998·1–3724·1	2086·3	1562·1–2784·3	1956·7	1483·9–2459·6	2618·4	2071·8–3217·5
Na (mg)*	1644·6	1135·6–2257·9	19 988·4	1353·9–2868·6	2375·5	1636·8–3338·7	2159·1	1466·3–3069·5
Zn (mg)†								
Mean	14·1		12·6		14·2		13·5	
sd	7·3		6·7		6·8		6·9	
Cu (mg)*	1·6	1·2–2·2	1·4	1·1–1·8	1·5	1·2–1·9	1·5	1·1–2·1
Mn (mg)†								
Mean	2426·8		2168·2		2358·7		2266·2	
sd	1537·1		1143·7		1000·5		1204·8	
Vitamin A (µg)*	1744·2	1158·1–2536·3	1393·3	858·8–2122·8	133·0	889·2–2073·2	1638·1	1058·6–2314·1
Thiamine (mg)*	2·0	1·6–2·5	1·5	1·2–2·0	1·8	1·4–2·3	1·9	1·4–2·5
Riboflavin (mg)*	1·7	1·2–2·6	1·6	1·2–2·1	1·6	1·2–2·3	1·6	1·1–2·5
Niacin (mg)*	26·0	19·1–36·4	21·7	16·0–28·6	23·7	18·1–30·6	25·6	21·1–32·5
Vitamin B6 (µg)*	3·8	2·8–5·3	3·3	2·2–4·2	2·8	2·0–3·8	4·1	3·2–4·9
Folate (µg)*	500·7	355·4–674·1	436·6	334·8–604·9	448·5	343·9–578·3	481·4	374·6–612·2
Vitamin B_12_ (µg) *	5·0	2·9–8·5	4·5	2·5–7·3	4·5	2·7–6·9	4·9	2·7–7·9
Pantothenic acid (mg)*	5·7	4·4–7·3	5·0	3·7–6·4	5·3	3·9–6·7	5·8	3·9–8·1
Biotin (µg)*	53·7	35·5–75·7	46·9	33·2–65·6	47·2	34·7–62·1	49·9	37·4–7
Vitamin C (mg)*	79·0	45·3–153·6	41·9	28·4–78·1	51·8	29·7–94·1	55·5	34·3–94·1
Vitamin D (mg)*	4·0	2·2–6·9	3·8	2·2–6·5	4·4	2·6–6·9	4·8	2·9–6·9
Vitamin E (mg)*	14·1	9·1–19·1	11·7	8·1–17·8	13·2	9·3–18·9	13·9	9·2–19·5

CHO; carbohydrate; n/a, not applicable.

* Non-parametric data presented as median (25th–75th percentiles).

† Parametric data presented as mean values and standard deviations.

‡ *P* value for significance of differences in nutrient value between each dietary pattern, comparing the highest tertile of each dietary pattern (Wilcoxon signed-rank test for non-parametric data and Paired *t* test for parametric data). Calculated as percentages, using each macro-nutrient’s energy (kJ/d), divided by total energy from total protein + carbohydrate + fat.

Online Supplementary Table S1 presents the top ten single foods consumed between breast cancer cases and controls within the unprocessed/minimally processed, processed and ultra-processed food groups. The culinary ingredients group was excluded from this table. Maize meal, white rice, apples, eggs and beetroot were among the most consumed single foods within the unprocessed/minimally processed food group in both breast cancer cases and controls.

Regarding processed foods, peanut butter, atchar (sweet and/or spiced condiment usually made from chillies, vegetables or mangoes), vetkoek (deep fried doughnut like dough) scones and canned pilchards (small fatty fish, also referred to as sardines) were among the top ten single foods consumed in both breast cancer cases and controls.

The most consumed single foods within the ultra-processed food group in both breast cancer cases and control participants were brown bread, white bread, soup powder, beef sausages (minced meat with salt, spices and other additives), margarine and carbonated soft drinks.

## Discussion

In this study of black women from Soweto, South Africa, consumption of unprocessed/minimally processed foods contributed to more than a third of the total EI per day in both case and control participants, while ultra-processed foods contributed to more than 44 % and processed food to about 12 % of the total EI per day. Higher consumption of unprocessed/minimally processed foods was inversely associated with breast cancer risk overall, for pre- and postmenopausal women, for PR+ breast cancers, for women with a BMI < 30 kg/m^2^ and for women with a BMI ≥ 30 kg/m^2^. No significant association with breast cancer risk was observed for higher consumption of culinary ingredients, processed and ultra-processed foods.

### Consumption of unprocessed/minimally processed foods and breast cancer risk

Unprocessed/minimally processed food consumption accounted for 38·8 % of total EI in breast cancer cases and 35·2 % in controls in this study. Studies investigating the association between breast cancer risk and unprocessed/minimally processed foods (NOVA 1), as a group, are lacking. However, similar intakes of unprocessed/minimally processed foods were observed in studies (investigating the association between unprocessed/minimally processed foods and risk of developing other non-communicable diseases or being obese) conducted in Canada (39·2 %), Australia (35 %) and Chile (33·8 %)^(15,36,37)^.

Results of our study are in line with other studies (conducted in different populations), which have found an inverse association between plant-based unprocessed/minimally processed food groups or dietary patterns with non-communicable diseases^(38,39)^. In general, plant-based unprocessed/minimally processed foods have a healthier nutrient profile with a lower energy density and high content of various phytochemicals (i.e. fibre, carotenoids and flavonoids), vitamins and minerals, which may be linked to several anti-cancerous cell effects in the human body^(3,37)^. The unprocessed/minimally processed food group in our study had a similar ‘healthy’ nutrient profile as these plant-based unprocessed/minimally processed foods, which may explain the significant inverse association thereof with breast cancer risk in our study. Unprocessed or minimally processed foods are also linked to a lower risk of gaining weight or being obese, which may reduce the risk of developing postmenopausal breast cancer^(3)^. Disease prevention dietary guidelines such as the World Cancer Research fund/American Institute for Cancer Research’s Cancer Prevention Recommendations and the South African Food Based Dietary Guidelines therefore advise to consume unprocessed whole foods (maize meal, fruits, vegetables, milk and lean meats) while limiting highly processed foods with added sugar, saturated fat and salt^(3,40)^. Results of this study support the use of the above-mentioned dietary guidelines as educational tools to promote healthy diets in Soweto, South Africa that could assist in preventing breast cancer development.

### Consumption of culinary ingredients and processed foods

The contribution of culinary ingredients and processed foods to total EI/d was similarly distributed in both breast cancer cases (6·1 and 10·9 %, respectively) and controls (5·5 and 12·8 %, respectively). Similar percentage energy contributions of culinary and processed foods to total EI/d were noted in other studies (Australia, UK, Canada and the USA)^(15,37,41–43)^. Higher consumption of culinary ingredients and processed foods (although very differently defined) did not show any significant association with breast cancer risk in our study. More research regarding culinary ingredients and processed food consumption is required in South Africa and in particular this population.

### Ultra-processed food consumption and breast cancer risk

Dietary contributions of ultra-processed foods to total EI per day in this study (> 44 %) were lower than studies conducted in the USA (between 55·0 and 57·5 %) and the UK (between 48·6 and 56·8 %)^(41–43)^. Our study showed similar percentage intakes of ultra-processed foods as in studies conducted in Canada (48 %) and Australia (42 %), while lower percentages of ultra-processed food consumption were noted in Belgium (29·6 %), France (18·7 %) and other middle-income countries such as Brazil (21·5 %), Mexico (29·8 %) and Chile (28·6 %)^(10,11,15,16,36,37,44)^. This indicates that this urban female population of Soweto, South Africa, an upper middle-income country, has higher percentages of ultra-processed food contributions in their diets than other middle-income countries and similar percentage intakes than high-income countries. However, cultural influences and preferences of different countries may also influence the amount of ultra-processed food consumed.

The high percentage ultra-processed food contributions to total EI in this study reflect the changes in dietary patterns due to the growing obesogenic food environment in South Africa^(5,45)^. In the South African context, cost and affordability can also be a major driver of food choices, irrespective of their nutritional value and quality^(46)^. For example, mass-produced fortified white-and-brown bread, an affordable staple food in South Africa, was the most consumed ultra-processed food item in both breast cancer cases and controls. Compared with affordable unprocessed/minimally processed starchy staples such as rice and maize meal in South Africa, mass-produced white-and-brown breads are cheaper and require less preparation time and may therefore be more frequently bought^(47)^.

Compared with the other NOVA food groups, the ultra-processed food group in our study had the highest content of saturated fat and added sugar while having the lowest content of dietary fibre. A diet consisting mainly of foods with a less healthy nutrient profile (high energy density and high content of added sugar, saturated fat and low intakes of fibre) reduces the overall quality of the diet and increases the risk of developing obesity and other diet-related non-communicable diseases^(12–14,40,41,46)^. The high percentage of ultra-processed food consumption in this study is therefore concerning and emphasises the need for healthy, affordable and sustainable food environments that prioritise nutrition policies supporting the NOURISHING framework^(47,48)^. However, creating healthier and affordable food environments remains a difficult task and will require commitment from various stakeholders across multiple (public, private and individual) sectors in South Africa^(49)^.

Interestingly, no significant associations were observed between higher ultra-processed food consumption and breast cancer risk in this study. In contrast to our findings, the French NutriNet-Santé cohort study (104 980 participants) showed that higher consumption of ultra-processed foods, using the NOVA system, was positively associated with breast cancer risk^(10)^. Another matched case–control study conducted in Brazil found that regular consumption of ultra-processed foods (> 5 portions per week) was associated with having a 2·35 times higher odds of developing breast cancer^(11)^. Although the ultra-processed food group in our study had a high content of energy, saturated fat and added sugar, together with a low content of dietary fibre, a high content of micronutrients was observed. The high content of micronutrients, which may increase the quality of the overall diet, may explain why no significant association between ultra-processed food consumption and breast cancer risk was observed in our study. It is therefore possible that the type and nutritional value of ultra-processed foods consumed in South Africa differ from the French and Brazilian studies. However, ultra-processed foods are not considered healthy since evidence has linked higher consumption thereof to various non-communicable diseases and therefore requires more research.

Given that cancer develops over an extended period, diet in the past several years may also influence cancer risk. Therefore, results of our study should be interpreted with caution since data collection of the SABC study focused on the participant’s current diet (past month at the time of diagnosis) and not past dietary intake of several years back. However, this is a general problem for any study that collect current dietary data only once. Additionally, it is possible that the period of data collection in our study overlapped with breast cancer symptoms, which could have led to changes in habitual dietary intake of breast cancer cases. Thus, a possibility of biased dietary data for cases due to changes in dietary intake, but dietary intake may also depend on the stage of cancer at diagnoses and therefore, require more research.

Our study has some strengths and limitations. Strengths include that cases were recruited prior to any breast cancer treatment and questionnaires used to obtain data were proven to be validated. Trained personnel were used to administer all questionnaires (not self-reported). Data used in the analysis were standardised and the categorisation of the food items according to the NOVA system was discussed with experts involved in the development of the NOVA system. Limitations include the relatively small sample size of this study, especially for subgroup analyses, and the fact that dietary intake was measured over the past month (at recruitment of the study) when habitual dietary intake of case participants could have changed due to illness (increased possibility for reverse causation, but may be dependent on the tumour size and requires more research). The nature of the study design could potentially result in non-differential misclassification and underestimation of the associations. In addition, although dietary intakes were captured throughout the year (in different participants) seasonal variability of foods (not adjusted for) may have influenced usual reporting of dietary intakes. The QFFQ used to evaluate the NOVA food groups was not specifically designed to capture foods based on their degree of processing. This made the recognition of each NOVA food group difficult. In addition, foods were categorised into their most usual form of consumption with the most conservative classification option (less processed level) chosen in a case of doubt. Information bias may therefore exist, while residual confounding can also not be ruled out.

In conclusion, unprocessed/minimally processed foods may reduce the risk of developing breast cancer in this population. Foods associated with unprocessed/minimally processed foods may play an important role in primary breast cancer prevention guidelines. However, more research on this topic is required and our results first need to be replicated in other South African regions and populations before any dietary guidelines can be formulated. Our results suggest that food environments and dietary intake behaviour should be prioritised to make unprocessed/minimally processed foods more affordable, accessible and available in South Africa. The high amount of EI from ultra-processed foods is worrisome as these foods may displace possibly protective foods from the unprocessed/minimally processed food group, which may reduce the risk of breast cancer in this population.

## Supplementary material

For supplementary material accompanying this paper visit https://doi.org/10.1017/S0007114522000423. click here to view supplementary material
