# Supplementary material for: Degree of food processing and breast cancer risk in black urban women from Soweto, South African: the South African Breast Cancer study
Source: Br J Nutr. 2022 Feb 3;128(11):2278–89. doi: 10.1017/S0007114522000423 (PMC9346100; doi:10.1017/S0007114522000423)
Supplement: Supplementary file 1 [file S0007114522000423sup001.docx]

**Supplementary Table 1 Top ten single foods consumed in the minimally/unprocessed and ultra-processed food groups in breast cases and controls.**

|  | **Controls (n=396)** | | **Breast cancer cases (n=396)** | |
| --- | --- | --- | --- | --- |
| **Unprocessed/ minimally processed foods** | **% of sample consuming product n (%)** | **†Energy**  **(kJ)** | **% of sample consuming product n (%)** | **†Energy**  **(kJ)** |
| Maize meal | 382 (96.5) | 770 (485; 1455) | 386 (97.5) | 970 (554; 1734) |
| White rice | 355 (89.6) | 239 (137; 531) | 345 (87.1) | 190 (99; 532) |
| Apple | 333 (84.1) | 243.2 (76; 266) | 331 (83.6) | 182 (61; 266) |
| Beetroot | 311 (78.5) | 49 (16; 286) | 292 (73.7) | 33 (16; 114) |
| Eggs | 307 (77.5) | 327 (109; 641) | 308 (77.8) | 227 (109; 616) |
| Banana | 282 (71.2) | 204 (60; 382) | 295 (74.5) | 120 (61; 382) |
| Full cream milk | 272 (68.7) | 262 (163; 281) | 259 (65.4) | 262 (144; 321) |
| Chicken | 255 (64.4) | 237 (118; 917) | 241 (60.1) | 236 (117; 917) |
| Pear | 250 (63.1) | 215 (69; 305) | 236 (59.6) | 144 (71; 300) |
| Potato | 175 (44.2) | 415 (112; 1243) | 152 (38.4) | 390 (89; 990) |
| **Processed foods** | **% of sample consuming product n (%)** | **Energy**  **(kJ)** | **% of sample consuming product n (%)** | **Energy**  **(kJ)** |
| Peanut butter | 195 (49.2) | 359.3 (104.8; 2620.0) | 219 (44.7) | 149.7 (74.9; 733.6) |
| Atchar | 182 (45.9) | 281 (45.7; 989.7) | 136 (34.3) | 112.3 (26.5; 712.6) |
| Vetkoek | 161 (40.7) | 696.7 (174.2; 1524.2) | 157 (39.7) | 522.5 (174.2; 1524.0) |
| Scone (plain) | 146 (36.9) | 686.6 (114.4; 1602.0) | 116 (29.9) | 228.9 (67.5; 1235.8) |
| Peanut butter | 137 (34.6) | 102.7 (34.2; 1198.2) | 123 (31.1) | 68.5 (17.1; 287.5) |
| Canned pilchards | 135 (34.1) | 74.6 (17.4; 455.8) | 100 (25.3) | 34.7 (17.6; 435.4) |
| Roasted/salted peanuts | 111 (28.0) | 185.3 (62.9; 459.2) | 123 (31.1) | 148.2 (62.9; 398.3) |
| Beer (4.5% alcohol) | 78 (19.7) | 389.5 (171.9; 1578.8) | 62 (15.7) | 229.1 (96.2; 1348.8) |
| Canned peaches | 77 (19.4) | 58.5 (22.5; 315.3) | 58 (14.7) | 25.9 (9.0; 81.0) |
| Canned vegetables | 62 (15.6) | 42.3 (21.4; 212.3) | 66 (16.6) | 38.2 (13.2; 201.4) |
| **Ultra-processed foods** | **% of sample consuming product n (%)** | **Energy**  **(kJ)** | **% of sample consuming product n (%)** | **Energy**  **(kJ)** |
| *Brown bread | 300 (75.8) | 444 (148; 1036) | 272 (68.7) | 222 (97; 932) |
| *White bread | 297 (75.0) | 1029 (617; 1275) | 299 (75.5) | 926 (529; 1234) |
| Soup powder | 291 (73.5) | 73 (28; 122) | 307 (77.5) | 66 (27; 183) |
| Beef sausage | 290 (73.2) | 278.4 (139.2; 1624.0) | 282 (71.2) | 278.6 (116.0; 835.2) |
| Margarine | 287 (72.5) | 595 (191; 2975) | 254 (64.1) | 405 (170; 1882) |
| Carbonated Soft drink | 234 (59.1) | 175 (87; 262) | 237 (59.8) | 175 (82; 263) |
| French Fries | 210 (53.0) | 218 (82; 1277) | 223 (56.3) | 218 (91; 912) |
| Crumbed Fish | 209 (52.8) | 158 (49; 854) | 180 (45.5) | 97 (44; 854) |
| Potato Crisps | 205 (51.8) | 238 (102; 2315) | 204 (51.5) | 238 (99; 833) |
| Tomato Sauce | 193 (48.7) | 82 (27; 480) | 164 (41.4) | 42 (20; 243) |

* fortified with vitamin A, thiamine, riboflavin, niacin, pyridoxine, folic acid, iron and zinc
† Reported as median (p 25^th^; p 75^th^)
