# Supplementary material for: Degree of food processing and breast cancer risk in black urban women from Soweto, South African: the South African Breast Cancer study
Source: Br J Nutr. Author manuscript; Available in PMC 2022 Dec 22. (PMC9346100; doi:10.1017/S0007114522000423)
Supplement: Supplementary file 1 [file NIHMS1793921-supplement-Supplementary_table_1.pdf]

Supplementary Table 1 Top ten single foods consumed in the minimally/unprocessed and ultra-processed food groups in breast cases and controls.

	Controls (n=396)		Breast cancer cases (n=396)	
**Unprocessed/ minimally processed foods**	**% of sample consuming product n (%)**	**†Energy (kJ)**	**% of sample consuming product n (%)**	**†Energy (kJ)**
Maize meal	382 (96.5)	770 (485; 1455)	386 (97.5)	970 (554; 1734)
White rice	355 (89.6)	239 (137; 531)	345 (87.1)	190 (99; 532)
Apple	333 (84.1)	243.2 (76; 266)	331 (83.6)	182 (61; 266)
Beetroot	311 (78.5)	49 (16; 286)	292 (73.7)	33 (16; 114)
Eggs	307 (77.5)	327 (109; 641)	308 (77.8)	227 (109; 616)
Banana	282 (71.2)	204 (60; 382)	295 (74.5)	120 (61; 382)
Full cream milk	272 (68.7)	262 (163; 281)	259 (65.4)	262 (144; 321)
Chicken	255 (64.4)	237 (118; 917)	241 (60.1)	236 (117; 917)
Pear	250 (63.1)	215 (69; 305)	236 (59.6)	144 (71; 300)
Potato	175 (44.2)	415 (112; 1243)	152 (38.4)	390 (89; 990)
**Processed foods**	**% of sample consuming product n (%)**	**Energy (kJ)**	**% of sample consuming product n (%)**	**Energy (kJ)**
Peanut butter	195 (49.2)	359.3 (104.8; 2620.0)	219 (44.7)	149.7 (74.9; 733.6)
Atchar	182 (45.9)	281 (45.7; 989.7)	136 (34.3)	112.3 (26.5; 712.6)
Vetkoek	161 (40.7)	696.7 (174.2; 1524.2)	157 (39.7)	522.5 (174.2; 1524.0)
Scone (plain)	146 (36.9)	686.6 (114.4; 1602.0)	116 (29.9)	228.9 (67.5; 1235.8)
Peanut butter	137 (34.6)	102.7 (34.2; 1198.2)	123 (31.1)	68.5 (17.1; 287.5)
Canned pilchards	135 (34.1)	74.6 (17.4; 455.8)	100 (25.3)	34.7 (17.6; 435.4)
Roasted/salted peanuts	111 (28.0)	185.3 (62.9; 459.2)	123 (31.1)	148.2 (62.9; 398.3)
Beer (4.5% alcohol)	78 (19.7)	389.5 (171.9; 1578.8)	62 (15.7)	229.1 (96.2; 1348.8)
Canned peaches	77 (19.4)	58.5 (22.5; 315.3)	58 (14.7)	25.9 (9.0; 81.0)
Canned vegetables	62 (15.6)	42.3 (21.4; 212.3)	66 (16.6)	38.2 (13.2; 201.4)
**Ultra-processed foods**	**% of sample consuming product n (%)**	**Energy (kJ)**	**% of sample consuming product n (%)**	**Energy (kJ)**
*Brown bread	300 (75.8)	444 (148; 1036)	272 (68.7)	222 (97; 932)
*White bread	297 (75.0)	1029 (617; 1275)	299 (75.5)	926 (529; 1234)
Soup powder	291 (73.5)	73 (28; 122)	307 (77.5)	66 (27; 183)
Beef sausage	290 (73.2)	278.4 (139.2; 1624.0)	282 (71.2)	278.6 (116.0; 835.2)
Margarine	287 (72.5)	595 (191; 2975)	254 (64.1)	405 (170; 1882)
Carbonated Soft drink	234 (59.1)	175 (87; 262)	237 (59.8)	175 (82; 263)
French Fries	210 (53.0)	218 (82; 1277)	223 (56.3)	218 (91; 912)
Crumbed Fish	209 (52.8)	158 (49; 854)	180 (45.5)	97 (44; 854)
Potato Crisps	205 (51.8)	238 (102; 2315)	204 (51.5)	238 (99; 833)
Tomato Sauce	193 (48.7)	82 (27; 480)	164 (41.4)	42 (20; 243)

* fortified with vitamin A, thiamine, riboflavin, niacin, pyridoxine, folic acid, iron and zinc

† Reported as median (p 25th; p 75th)
